# Human resources health supply chains and access to essential medicines

**DOI:** 10.1186/2052-3211-7-S1-I2

**Published:** 2014-12-17

**Authors:** Andrew Brown, Muhammad Atif, Erin Hasselberg, Pamela Steele, Chris Wright, Zaheer-Ud-Din Babar

**Affiliations:** 1 People that Deliver, UNICEF Supply Division, Copenhagen, Denmark; 2 Faculty of Pharmacy and Alternative Medicine, Islamia University, Bahawalpur, Pakistan; 3 John Snow Incorporated, Boston, MA, USA; 4 John Snow Incorporated, Addis Ababa, Ethiopia; 5 Pamela Steel Associates Ltd, Oxford, United Kingdom; 6 School of Pharmacy, University of Auckland, Auckland, New Zealand

## 

With up to a third of the world’s population with limited access to essential medicines, it is clear that by 2015 many countries will not be able to achieve their health related Millennium Development Goals (MDGs) [1]. Of the eight MDGs, four explicitly discusses the availability of medicines at the primary care or service delivery point level [2]. It is pertinent because without access to and appropriate use of quality medicines, health systems would lose their ability to meet healthcare needs.

Though affordability of medicines and high prices are frequently highlighted as challenges to access to essential medicines, the weakness of health supply chains has remained a consistent barrier across a range of low and middle -income countries [3-5]. Despite major investment over the past decades, national supply chains are often unable to respond effectively to existing demands, putting health outcomes at risk. Since the first Global Forum on Human Resources for Health in Kampala in 2008 [6], the human resource focus has been on the doctors, nurses, midwives and community health workers. However, there is little focus on human resources to improve and sustain health supply chains.

A focus on the human resources is needed and in this context, in 2011, the People that Deliver (PtD) Initiative was founded. The International Pharmacy Federation (FIP) provided further evidence of the need for a HR focus in SCM through their Global Workforce Report in 2012 [7]. In that report they make a link between a lack of pharmacy personnel and inequalities in access to medicines. For example in Sub-Saharan Africa, on average less than one pharmacist was observed for 10,000 population [8]. In October 2014 the 2^nd^ Global Conference on Human Resources for Supply Chain Management (SCM) was held to demonstrate the achievement PtD has made in the recent years [http://www.peoplethatdeliver.org].

Launched in 2011, the PtD Initiative is a global partnership of over 80 organizations who have the joint vision of a world where an agenda for national health supply chain workforce is developed. (http://www.peoplethatdeliver.org). Specifically the goals of PtD are:

I. Global recognition that strong supply chains are essential for positive health outcomes and require a competent, recognized and supported supply chain workforce with significant technical and managerial capacity.

II. Government and national health institutions demand, recruit and retain appropriately qualified personnel for positions with supply chain responsibilities.

III. Adequate personnel from relevant cadres with appropriate supply chain competencies and qualifications are available.

IV. A repository of evidence-based resources for HR for SCM is established, accessible, used and disseminated.

Human resources are a key performance driver within public health supply chains. The effective management of a supply chain demands excellence in managing its human resources, an area particularly overlooked in resource poor environments. By proactively managing plans, policies and procedures associated with people, an organisation can improve supply chain performance. Such a systematic approach requires the need to plan, finance, develop, support, and retain the national workforces needed for the effective, efficient, and sustainable management of health supply chains [9,10].

The 2nd PtD Global Conference on Human Resources in Supply Chain Management Conference presented international and country-based work around five interrelated sub themes (Table 1):

**Table 1:** The five sub themes of the 2^nd^ PtD Global Conference on HR for SCM

Assessment and planning	Assessing HR systems, creating policies, plans and standard operating procedures for an effective and sustainable SCM workforce.
**Leaders and change agents**	Engaging powerful stakeholders and SCM leaders to put HR for SCM on the agenda and enact local change.

**Workforce development**	Developing the SCM workforce through contextualised pre-service education and continued professional development.

**Increasing performance**	Increasing the performance and retention of SCM personnel within an organisational context.

**Professionalization**	Improving education approaches for health logistics and supply chain personnel, and growing professional communities.

The abstracts presented in this special issue highlight current global activity in this area and lay the foundation for the second phase of PtD 2015-2016. Some of the themes presented in the conference include, the increasing use of the HR for SCM assessment tool, application of SCM competency modelling, varied approaches to SCM workforce development, and local professionalization activities.

As the post 2015 development agenda moves its focus toward health equity, the world’s increasing population and expanding middle class will place even greater demands on health services. These increasing demands will put further strain on the health supply chains needed to provide these services. In resource constrained environments, the challenge will be to provide a business case to governments, convincing them of the need to invest in health supply chains. The international development agenda will require organisations involved in health supply chains to come together in a more coordinated fashion, working with governments to enact local, sustainable change. The People that Deliver Initiative will continue to provide a platform to ensure that HR for SCM remains on the international agenda.
